# The combination of MLN2238 (ixazomib) with interferon-alpha results in enhanced cell death in melanoma

**DOI:** 10.18632/oncotarget.12791

**Published:** 2016-10-21

**Authors:** Lorena P. Suarez, Gregory M. Kemper, Megan C. Duggan, Andrew Stiff, Tiffany C. Noel, Joseph Markowitz, Eric A. Luedke, Vedat O. Yildiz, Lianbo Yu, Alena Cristina Jaime-Ramirez, Volodymyr Karpa, Xiaoli Zhang, William E. Carson

**Affiliations:** ^1^ The Arthur G. James Comprehensive Cancer Center and Richard J. Solove Research Institute, The Ohio State University, Columbus, OH, USA; ^2^ Biomedical Sciences Graduate Program, The Ohio State University, Columbus, OH, USA; ^3^ Division of Medical Oncology, Department of Internal Medicine, The Ohio State University, Arthur G. James Cancer Hospital and Richard J. Solove Research Institute, Columbus OH, USA; ^4^ Division of Surgical Oncology, Department of Surgery, The Ohio State University, Columbus OH, USA; ^5^ Center for Biostatistics, Department of Biomedical Informatics, The Ohio State University, Columbus, OH, USA

**Keywords:** MLN2238, ixazomib, melanoma, proteasome inhibitor, interferon-alpha

## Abstract

The ubiquitin-proteasome signaling pathway is critical for cell cycle regulation and neoplastic growth. Proteasome inhibition can activate apoptotic pathways. Bortezomib, a selective proteasome inhibitor, has anti-melanoma activity. MLN2238 (ixazomib), an oral proteasome inhibitor, has improved pharmacotherapeutic parameters compared to bortezomib. Interferon-alpha (IFN-α), an immune boosting agent, is FDA-approved for treatment of melanoma. In this study *in vitro* and *in vivo* evaluation of the antitumor potential of ixazomib and combination treatments with ixazomib and IFN-α were performed. Apoptosis induced by ixazomib was first observed at 12 hours and was maximal at 48 hours with similar levels of cell death compared to bortezomib. IFN-α alone had little effect on cell viability *in vitro*. However, the combination of ixazomib with IFN-α significantly enhanced ixazomib's ability to induce apoptotic cell death in BRAF V600E mutant and BRAF wild-type human melanoma tumor cells. The combination of ixazomib and IFN-α also enhanced inhibition of cell proliferation in BRAF V600E mutant melanoma tumor cells; however, this was not seen in BRAF wild-type cells. Ixazomib-induced apoptosis was associated with processing of the pro-apoptotic proteins procaspase-3, -7, -8, and -9, and cleavage of poly-ADP-ribose polymerase (PARP). In an *in vivo* xenograft model of human melanoma, combination treatment with IFN-α-2b and ixazomib demonstrated a significant reduction in tumor volume when compared to vehicle (p = 0.005) and single therapy ixazomib (p = 0.017) and IFN-α-2b (p = 0.036). These pre-clinical results support further evaluation of combination treatment with ixazomib and IFN-α for the treatment of advanced BRAF V600E mutant melanoma.

## INTRODUCTION

Melanoma is the deadliest skin cancer, and in 2015 it is estimated that there will be over 73,800 new cases of melanoma in the United States and almost 10,000 deaths [1]. The ubiquitin-proteasome signaling (UPS) pathway plays a critical role in cell cycle regulation, neoplastic growth, and metastatsis [2]. The proteasome is crucial for cellular regulation of protein synthesis and degradation and its proper functioning is essential to cell viability [3]. Loss of regulation in the UPS pathway has been linked to the pathogenesis of various malignancies and therefore represents a potential therapeutic target. Malignant cells are more dependent on removal of misfolded or damaged proteins by the proteasome secondary to their genetic instability and rapid proliferation [3]. Proteasome inhibition in malignant cells results in the stabilization and accumulation of these proteins, which leads to the activation of anti-proliferative signals, cell cycle disruption, activation of apoptotic pathways and cell death [3, 4].

Bortezomib is an intravenously administered selective inhibitor of the 26S proteasome subunit [5]. Bortezomib was the first FDA approved proteasome inhibitor and has demonstrated considerable apoptotic and anti-tumor activity in a variety of tumor cell lines, animal models, and clinical trials for advanced multiple myeloma, non-Hodgkin's lymphoma, mantle cell lymphoma, and non-small cell lung cancer [3, 4, 5]. Several studies have evaluated the efficacy of bortezomib in the treatment of melanoma. However, a phase II trial of bortezomib for the treatment of metastatic melanoma did not demonstrate a positive clinical outcome [2]. Bortezomib has shown minimal activity against melanoma (NCT00288041, NCT00580320, NCT01462773, and NCT01078961) [2, 6, 7, 8, 9, 10], but this may be secondary to its long proteasome dissociation half-life resulting in a slow dissociation from red blood cells and slow tumor entry [9].

MLN2238 (ixazomib) is a novel selective inhibitor of the 20S proteasome subunit and can be administered intravenously and orally [11, 12]. The distribution profiles after intravenous and oral dosing of ixazomib are similar. Ixazomib has been shown to have an oral bioavailability of 60% based on pooled population pharmacokinetic data analysis of intravenous and oral regimens [13]. Pharmacokinetic parameters of ixazomib are not affected by body-surface area, creatinine clearance, gender, or age; important features for dose simplification and potential future clinical use [13]. Preclinical studies have demonstrated that ixazomib has antitumor activity similar to that of bortezomib [11, 14, 15]. These studies have also demonstrated increased proteasome inhibition and improved pharmacokinetic and pharmacodynamic parameters for ixazomib [11, 14, 15]. Additionally, ixazomib has been demonstrated to have a six-fold faster proteasome disassociation half-life compared to bortezomib, resulting in a faster dissociation from red blood cells and more rapid tumor entry [11, 17].

Several phase I studies have evaluated the safety profile of ixazomib. Phase I studies evaluating intravenous ixazomib in patients with relapsed/refractory lymphoma and advanced non-hematologic malignancies with once-weekly and twice-weekly dosing, respectively, demonstrated that the drug was well tolerated with a manageable toxicity profile and durable antitumor activity in heavily pretreated patients, despite their prior exposure to multiple chemotherapeutic regimens (NCT00893464 and NCT00830869) [16, 17]. Two phase I studies of oral ixazomib (one with once-weekly dosing and the other with twice-weekly dosing) in patients with relapsed/refractory multiple myeloma also demonstrated drug tolerability of oral ixazomib with a manageable toxicity profile and durable antitumor activity (NCT00963820 and NCT00932698) [18, 19]. A phase II trial found ixazomib to be a promising agent in patients with relapsed multiple myeloma with, again, a favorable toxicity profile (NCT01415882) [20].

Recombinant interferon-alpha (IFN-α) has been used in the treatment of melanoma and renal cell carcinoma and has been associated with regression of metastatic disease [4, 21, 22]. Currently, IFN-α is FDA approved for adjuvant therapy in patients with Stage IIB-IIIC melanoma. The main mechanism by which IFN-α induces its anti-tumor effect in the treatment of melanoma is through its immunostimulatory properties and activation of the innate immune system's anti-tumor functions. However, IFN-α has been shown to sensitize malignant cells to apoptotic stimuli by increasing the expression of cell cycle regulatory proteins and proteins involved in the death receptor cascade [4]. Proteasome inhibition results in the stabilization and continued excess accumulation of cell cycle proteins, which lead to the activation of apoptotic pathways and cell death. Previous studies from our group have demonstrated that combination treatment with bortezomib and IFN-α synergistically enhances melanoma apoptotic cell death through the Fas Associated Death Domain (FADD)-induced caspase-8 activation [4].

The improved pharmacokinetic and pharmacodynamic parameters of ixazomib compared to bortezomib have made it a focus of investigation for use in combination with other pro-apoptotic agents [11, 14]. The purpose of the present study was to examine the antitumor potential of single-agent ixazomib, compare it to bortezomib and evaluate ixazomib treatment combinations with IFN-α *in vitro* and *in vivo*. We hypothesized that ixazomib would induce apoptosis in human melanoma cells and that IFN-α would enhance its apoptotic and anti-tumor activity. A secondary aim was to evaluate the usefulness of this combination in BRAF V600E mutant compared to BRAF wild-type melanoma cell lines.

## RESULTS

### Treatment of BRAF V600E mutant human melanoma tumor cells with ixazomib leads to apoptotic cell death

**Figure 1:**
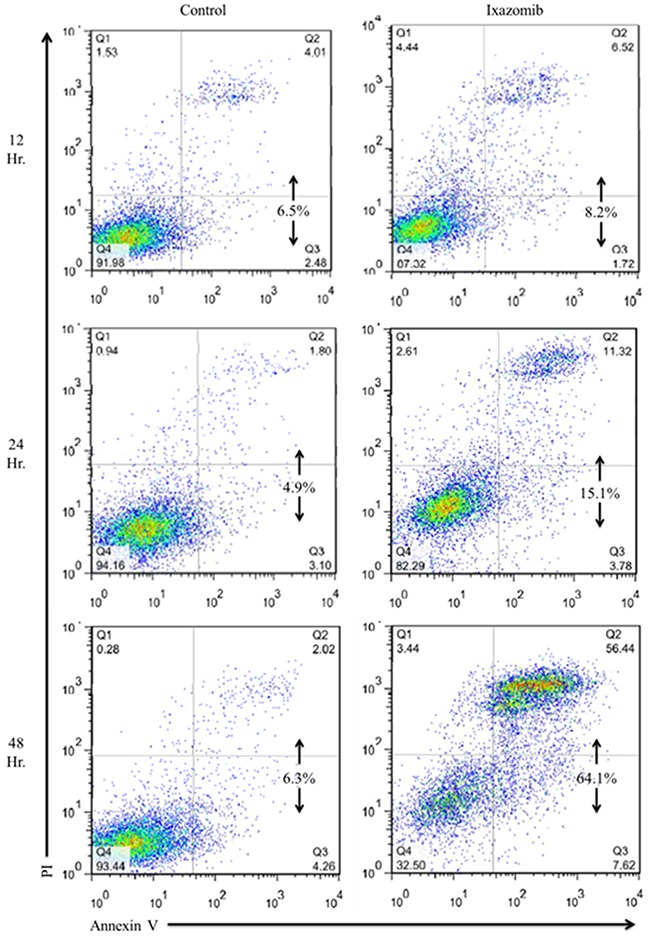
Ixazomib induces apoptosis in human BRAF V600E mutant (A375) cell line with maximal levels reached at 48 hours A375 (BRAF V600E mutant) human melanoma tumor cells plated at a density of 2 × 10^5^ cells/well on a 6 well plate were treated with complete medium supplemented with either 10% dimethyl sulfoxide (control) or 35 nM ixazomib. Cells were incubated for 12, 24 or 48 hours. The cells were subjected to annexin V/propidium iodide staining and flow cytometric analysis to determine levels of apoptosis.

The ability of ixazomib to induce apoptosis of BRAF V600E mutant human melanoma cells was evaluated via annexin V/ propidium iodide (PI) staining and flow cytometric analysis. The A375 (BRAF V600E mutant) human melanoma tumor cell line was used and cells were plated on a six well plate at a density of 2 × 10^5^ cells/well. Cells were then treated with complete medium supplemented with either 10% dimethyl sulfoxide (DMSO; control) or 35 nM ixazomib. Time course experiments were conducted and levels of apoptosis were measured after cells were incubated for 12, 24, and 48 hours (Figure 1). The induction of apoptosis began at 12 hours and reached maximal levels at approximately 48 hours with 65.1 ± 4.3% (mean ± standard error of the mean) cell death. Given this finding, 48 hours was established as the optimal incubation time for the remaining experiments.

**Figure 2:**
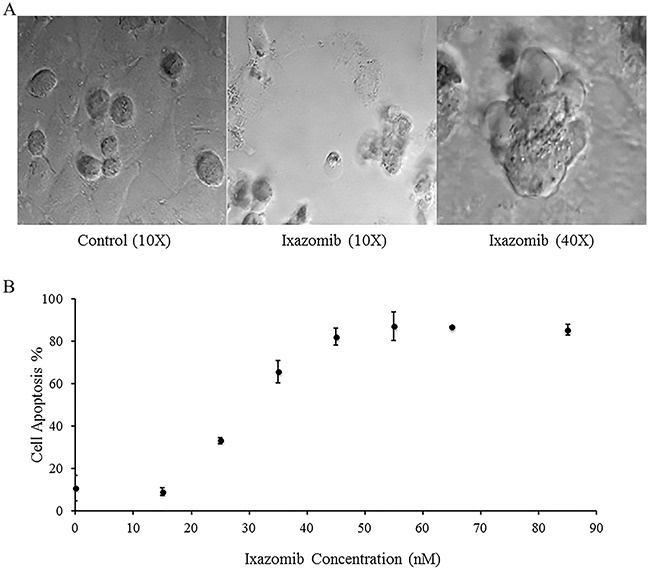
Treatment of BRAF V600E mutant human melanoma cell lines with ixazomib leads to apoptotic cell death with half maximal effective concentration (EC_50_) of 28.8 nM A375 (BRAF V600E mutant) human melanoma tumor cells were plated on a 6 well plate at a density of 2 × 10^5^ cells/well. **A.** A375 cells were treated with complete medium supplemented with either 10% dimethyl sulfoxide (control) or with 65 nM ixazomib. Differential interference contrast images were obtained using an Olympus Fluoview 1000MPE confocal microscope after 48 hour incubation. **B.** A375 cells were treated with complete medium supplemented with either 10% dimethyl sulfoxide (control, 0 nM ixazomib dose) or with varying doses of ixazomib for 48 hours. After incubation, the cells were subjected to annexin V/propidium iodide staining and flow cytometric analysis to determine the levels of apoptosis. Data represented as mean ± standard error of the mean.

A375 human melanoma tumor cells were plated on a six well plate at a density of 2 × 10^5^ cells/well and treated with complete medium supplemented with either 10% DMSO or 65 nM ixazomib. Differential interference contrast images of the A375 cells were obtained using an Olympus Fluoview 1000MPE confocal microscope following 48 hour ixazomib treatment (Figure 2A). Microscopic evaluation demonstrated histologic evidence of apoptotic cell death (blebs and reduced cell volume) as compared to the control group. A375 cells were then treated for 48 hours with complete medium supplemented with either 10% DMSO or varying doses of ixazomib (15, 25, 35, 45, 55, 65, and 85 nM). The levels of apoptosis were measured by annexin V/PI staining and flow cytometric analysis (Figure 2B). The half maximal effective concentration (EC_50_) dose of ixazomib-induced apoptotic cell death for A375 melanoma cells was calculated to be 28.8 nM (95% CI, 26.7-30.8).

### Treatment of the BRAF wild-type human melanoma tumor cells with ixazomib also leads to apoptotic cell death

**Figure 3:**
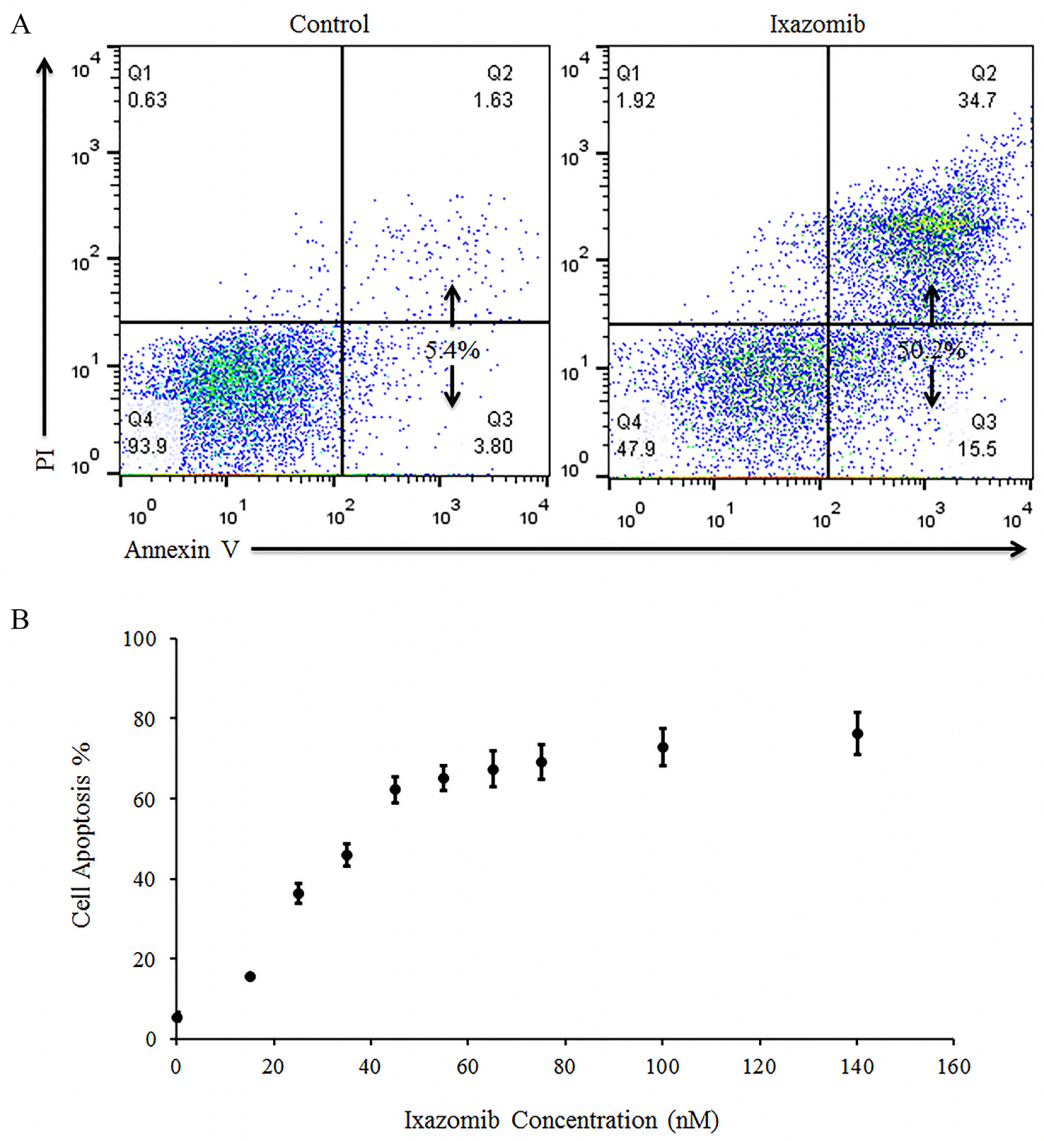
Treatment of BRAF wild-type human melanoma cell lines with ixazomib leads to apoptotic cell death with half maximal effective concentration (EC_50_) of 26.9 nM WM1366 (BRAF wild-type) human melanoma tumor cells were plated on a 6 well plate at a density of 2 × 10^5^ cells/well. **A.** WM1366 cells were treated for 48 hours with complete medium supplemented with either 10% dimethyl sulfoxide (control) or with 35 nM ixazomib. Cells were then subjected to annexin V/propidium iodide (PI) staining and flow cytometric analysis to determine the levels of apoptosis. **B.** WM1366 cells were treated for 48 hours with complete medium supplemented with either 10% dimethyl sulfoxide (control, 0 nM ixazomib dose) or with varying doses of ixazomib. After incubation, the cells were subjected to annexin V/PI staining and flow cytometric analysis to determine the levels of apoptosis. Data represented as mean ± standard error of the mean.

The ability of ixazomib to induce apoptosis of BRAF wild-type human melanoma cells was evaluated. The WM1366 (BRAF wild-type) human melanoma tumor cell line was used and cells were plated on a six well plate at a density of 2 × 10^5^ cells/well. Cells were then treated with complete medium supplemented with either 10% DMSO or 35 nM ixazomib. Time course experiments were conducted and levels of apoptosis were measured after cells were incubated for 12-48 hours via annexin V/PI staining and flow cytometric analysis. Maximal cell death, with an average of 47.4 ± 2.6% apoptosis, was seen at 48 hours (Figure 3A). WM1366 cells were then treated for 48 hours with complete medium supplemented with either 10% DMSO or varying doses of ixazomib. The levels of apoptosis were measured by annexin V/PI staining and flow cytometric analysis (Figure 3B). The EC_50_ dose of ixazomib-induced apoptotic cell death for WM1366 melanoma cells was calculated to be 26.9 nM (95% CI, 16.5-37.3).

### Treatment of BRAF V600E mutant human melanoma tumor cells with ixazomib results in reduced cell proliferation

**Figure 4:**
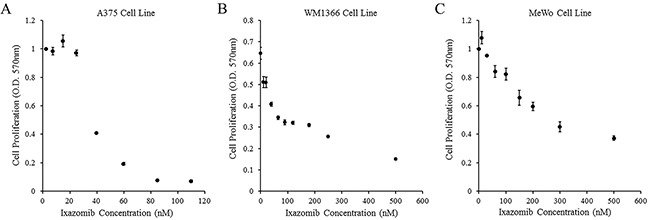
Treatment of human melanoma cell lines with ixazomib results in reduced tumor cell proliferation BRAF V600E mutant (A375) and BRAF wild-type (WM1366 and MeWo) human melanoma tumor cells were plated at a density of 3 × 10^4^ cells/well in 96 well plates and treated with complete medium supplemented with either 10% dimethyl sulfoxide (control, 0 nM ixazomib dose) or various concentrations of ixazomib for 48 hours. After incubation, methylthiazolyldiphenyl-tetrazolium bromide (MTT) cell proliferation assays were performed and cell proliferation rates were measured as optical densities (O.D.) at 570 nm. Data represented as mean ± standard error of the mean. **A.** A375 human melanoma tumor cell line. **B.** WM1366 human melanoma tumor cell line. **C.** MeWo human melanoma tumor cell line.

The ability of ixazomib to inhibit cell proliferation of BRAF V600E mutant human melanoma was evaluated. The A375 (BRAF V600E mutant) human melanoma tumor cell line was used and cells were plated at a density of 3 × 10^4^ cells/well in a 96 well plate. Cells were then treated with complete medium supplemented with either 10% DMSO or various concentrations of ixazomib for 48 hours. Cell proliferation following treatment with ixazomib was evaluated using methylthiazolyldiphenyl-tetrazolium bromide (MTT) cell proliferation assay and optical density (O.D.) recorded at a 570 nm wavelength using a microtiter plate reader (Figure 4A). The half maximal inhibitory concentration (IC_50_) dose for cell proliferation of ixazomib in A375 melanoma cells was calculated to be 36.7 nM.

### Treatment of BRAF wild-type human melanoma tumor cells with ixazomib also results in reduced cell proliferation

The ability of ixazomib to inhibit cell proliferation of BRAF wild-type human melanoma cells was evaluated. BRAF wild-type (WM1366 and MeWo) human melanoma tumor cell lines were used and cells were plated at a density of 3 × 10^4^ cells/well in 96 well plates. Cells were then treated with complete medium supplemented with either 10% DMSO or various concentrations of ixazomib for 48 hours. Cell proliferation following treatment with ixazomib was evaluated using MTT cell proliferation assay as described above (Figure 4B & 4C). The IC_50_ doses for cell proliferation of ixazomib in WM1366 and MeWo melanoma cells were calculated to be 33.5 nM and 320.8 nM, respectively. The IC_50_ dose of the MeWo cell line was much higher than the maximum tolerated oral dose of 170 nM demonstrated in previous phase I clinical trials (NCT00963820 and NCT00932698) [18, 19]. Therefore, a 35 nM dose of ixazomib was utilized for the rest of this study due to its proximity to the EC_50_ and IC_50_ doses of A375 and WM1366 cell lines.

### Proteasome inhibition with ixazomib results in similar levels of apoptotic cell death compared with bortezomib in human melanoma tumor cells

Human melanoma tumor cells (A375 and WM1366) were plated at a density of 2 × 10^5^ cells/well on 6 well plates and treated with complete medium supplemented with either 35 nM ixazomib or 10 nM bortezomib for 24 or 48 hours. Cells were then collected and resuspended in serum-free medium. An aliquot of cells was used for 1:1 staining with 0.4% trypan blue and the number of viable and dead cells were calculated using a hemocytometer. Treatment with ixazomib and bortezomib resulted in similar levels of cell death as measured by trypan blue staining (data not shown).

**Figure 5:**
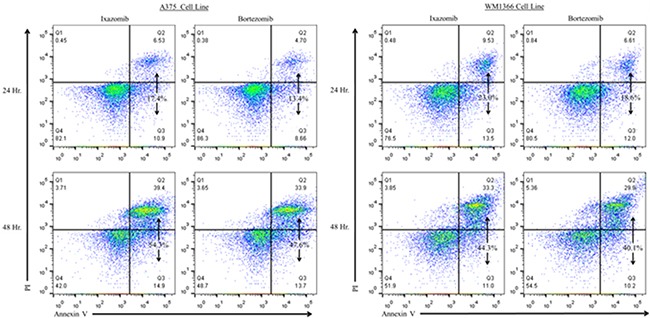
Proteasome inhibition with ixazomib results in similar levels of tumor cell apoptosis compared with bortezomib in human melanoma cell lines BRAF V600E mutant (A375) and BRAF wild-type (WM1366) human melanoma tumor cells plated at a density of 2 × 10^5^ cells/well on 6 well plates were treated for 24 or 48 hours with complete medium supplemented with either 35 nM ixazomib or 10 nM bortezomib. After incubation, the cells were subjected to annexin V/propidium iodide staining and flow cytometric analysis to determine the levels of apoptosis.

The levels of apoptosis were also evaluated using annexin V/PI staining and flow cytometric analysis (Figure 5). Twenty-four hour treatment of the A375 (BRAF V600E mutant) and WM1366 (BRAF wild-type) cell lines with 35 nM ixazomib resulted in 17.4% and 23.0% cell death, respectively, compared to 13.4% and 18.6% cell death when treated with 10 nM bortezomib. After 48 hours, A375 and WM1366 cell lines treated with 35 nM ixazomib exhibited 54.3% and 44.3% cell death, respectively, compared to 47.6% and 40.1% cell death when treated with 10 nM bortezomib. Although ixazomib has different binding kinetics from bortezomib, inhibition of the proteasome with ixazomib results in similar levels of apoptotic cell death.

### Combined treatment with ixazomib and IFN-α results in enhanced apoptotic cell death in BRAF V600E mutant and BRAF wild-type human melanoma tumor cells

**Figure 6:**
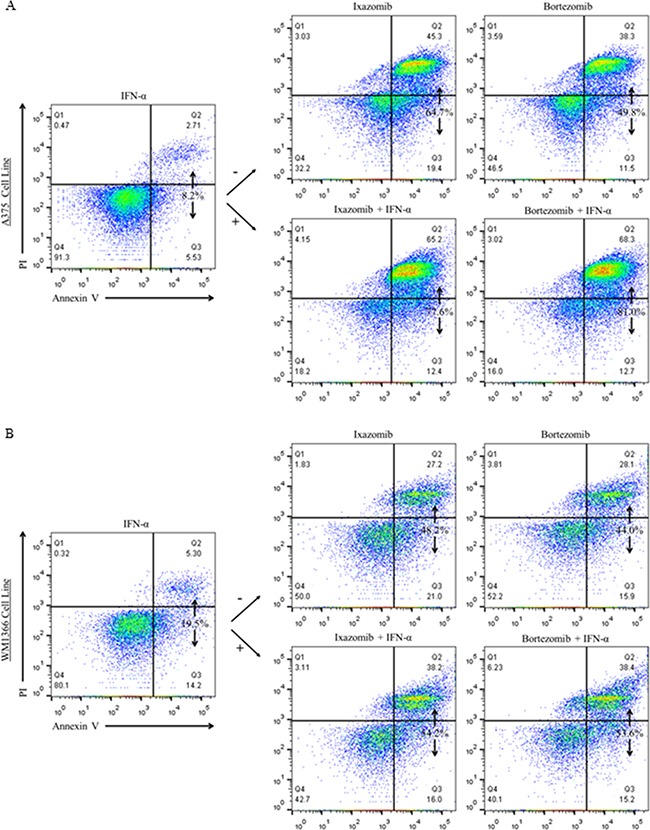
Addition of IFN-α to treatment of melanoma cell lines with proteasome inhibitor results in enhanced tumor cell apoptosis BRAF V600E mutant (A375) and BRAF wild-type (WM1366) human melanoma cells plated at a density of 2 × 10^5^ cells/well on 6 well plates were treated for 48 hours with complete medium supplemented with either 5 × 10^3^ U/mL IFN-α, 35 nM ixazomib, 10 nM bortezomib, or with the combination of ixazomib or bortezomib plus IFN-α. After 48 hour incubation, the cells were subjected to annexin V/propidium iodide staining and flow cytometric analysis to determine the levels of apoptosis.

Human melanoma tumor cells (A375 and WM1366) were plated at a density of 2 × 10^5^ cells/well on 6 well plates and treated for 48 hours with complete medium supplemented with either 5 × 10^3^ U/mL IFN-α, 35 nM ixazomib, 10 nM bortezomib, or the combination of ixazomib or bortezomib plus IFN-α. The levels of apoptosis were then measured using annexin V/PI staining and flow cytometric analysis (Figure 6). Single agent 48 hour treatment of IFN-α resulted in a minimal increase in apoptotic cell death in both BRAF V600E mutant (A375) and BRAF wild-type (WM1366) human melanoma tumor cells (4.9 ± 1.8 % and 12.5 ± 0.8%, respectively). Forty-eight hour treatment with ixazomib plus IFN-α resulted in increased apoptotic cell death compared to treatment with ixazomib alone with an observed level of apoptosis of 74.1 ± 5.1% vs. 53.6 ± 2.0% (p = 0.002) in A375 cells and 59.8 ± 0.3% vs. 49.7 ± 0.5% (p < 0.000) in WM1366 cells, respectively. Similarly, 48 hour treatment with bortezomib plus IFN-α resulted in increased apoptotic cell death compared to treatment with bortezomib alone with an observed level of apoptosis of 80.8 ± 1.45% vs. 57.3 ± 0.3% (p = 0.001) in A375 cells and 60.5 ± 1.8% vs. 48.2 ± 1.4% (p = 0.002) in WM1366 cells, respectively.

A linear mixed effects model was employed to assess whether treatment with ixazomib or bortezomib plus IFN-α induced synergistic apoptosis. Similar to previous studies [4], the analysis of A375 and WM1366 cell lines treated with bortezomib plus IFN-α revealed a significant synergistic apoptotic effect between the two treatments (p = 0.003 and p = 0.028, respectively). Analysis of A375 and WM1366 cell lines treated with ixazomib plus IFN-α also revealed a significant synergistic apoptotic effect between the two treatments (p = 0.011 and p = 0.007, respectively).

### Combined treatment with ixazomib and IFN-α results in decreased cell proliferation in BRAF V600E mutant human melanoma tumor cells

**Figure 7:**
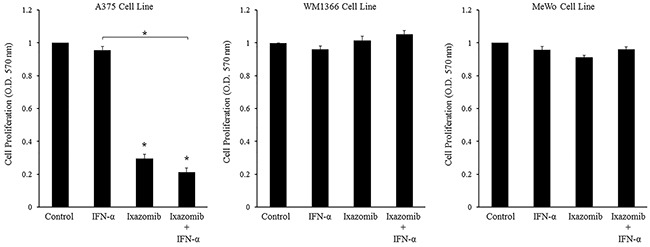
Ixazomib in combination with IFN-α results in reduced tumor cell proliferation in BRAF V600E mutant human melanoma cells BRAF V600E mutant (A375) and BRAF wild-type (WM1366 and MeWo) human melanoma tumor cells plated at a density of 3 × 10^4^ cells/well in 96 well plates were treated with complete medium supplemented with either 10% dimethyl sulfoxide (control), 5 × 10^3^ U/mL IFN-α, 35 nM ixazomib or the combination of 35 nM ixazomib plus IFN-α for 48 hours. After incubation methylthiazolyldiphenyl-tetrazolium bromide (MTT) cell proliferation assay was then performed and cell proliferation rates were measured as optical densities (O.D.) at 570 nm. Data represented as mean ± standard error of the mean. Statistical analysis was performed using t-statistics (* = p < 0.05 vs. controls and * with underlying bracket = p < 0.05 for comparisons among groups).

BRAF V600E mutant (A375) and BRAF wild-type (WM1366 and MeWo) human melanoma tumor cells were plated at a density of 3 × 10^4^ cells/well in 96 well plates and treated with complete medium supplemented with either 10% DMSO, 35 nM ixazomib, 10^3^ U/mL IFN-α, or the combination of ixazomib plus IFN-α for 48 hours. Cell proliferation was then evaluated using MTT cell proliferation assay and O.D. recorded at a 570 nm wavelength using a microtiter plate reader (Figure 7).

Evaluation of the treatment effects in the A375 cell line was performed. Single agent treatment with IFN-α did not lead to a significant decrease in cell proliferation compared to the control. Single agent treatment with ixazomib resulted in a significant decrease in cell proliferation when compared to the control (0.331 ± 0.01 vs. 1.00 ± 0.00 O.D., respectively, p < 0.000). Combination treatment with ixazomib plus IFN-α resulted in a significant decrease in cell proliferation when compared to the control group (0.198 ± 0.02 vs. 1.00 ± 0.00 O.D., respectively, p < 0.000) and a larger decrease in cell proliferation than either agent alone; however, this decrease in cell proliferation was only found to be significant between the IFN-α vs. ixazomib plus IFN-α treatment groups (1.016 ± 0.02 vs. 0.198 ± 0.02 O.D., respectively, p < 0.000). A linear mixed effects model was employed to assess whether treatment with ixazomib plus IFN-α resulted in synergistic inhibition of cell proliferation. However, this relationship was determined to be non-synergistic (p = 0.958).

These same evaluations were performed in the two different BRAF wild-type human melanoma cell lines, WM1366 and MeWo. Single agent treatment with IFN-α did not demonstrate a significant decrease in cell proliferation compared to the control in either of the two cell lines. Both the WM1366 and the MeWo cell lines were more resistant to ixazomib treatment, as measured by the MTT proliferation assay, with no significant inhibition of cell proliferation from ixazomib or ixazomib plus IFN-α treatments.

Similar results were observed in all three cell lines when comparing the treatments of 5 × 10^3^ U/mL IFN-α, 10 nM bortezomib, and the combination of bortezomib plus IFN-α (data not shown).

### Ixazomib therapy induces processing of effector caspases and poly (ADP-ribose) polymerase (PARP) in human melanoma tumor cells

**Figure 8:**
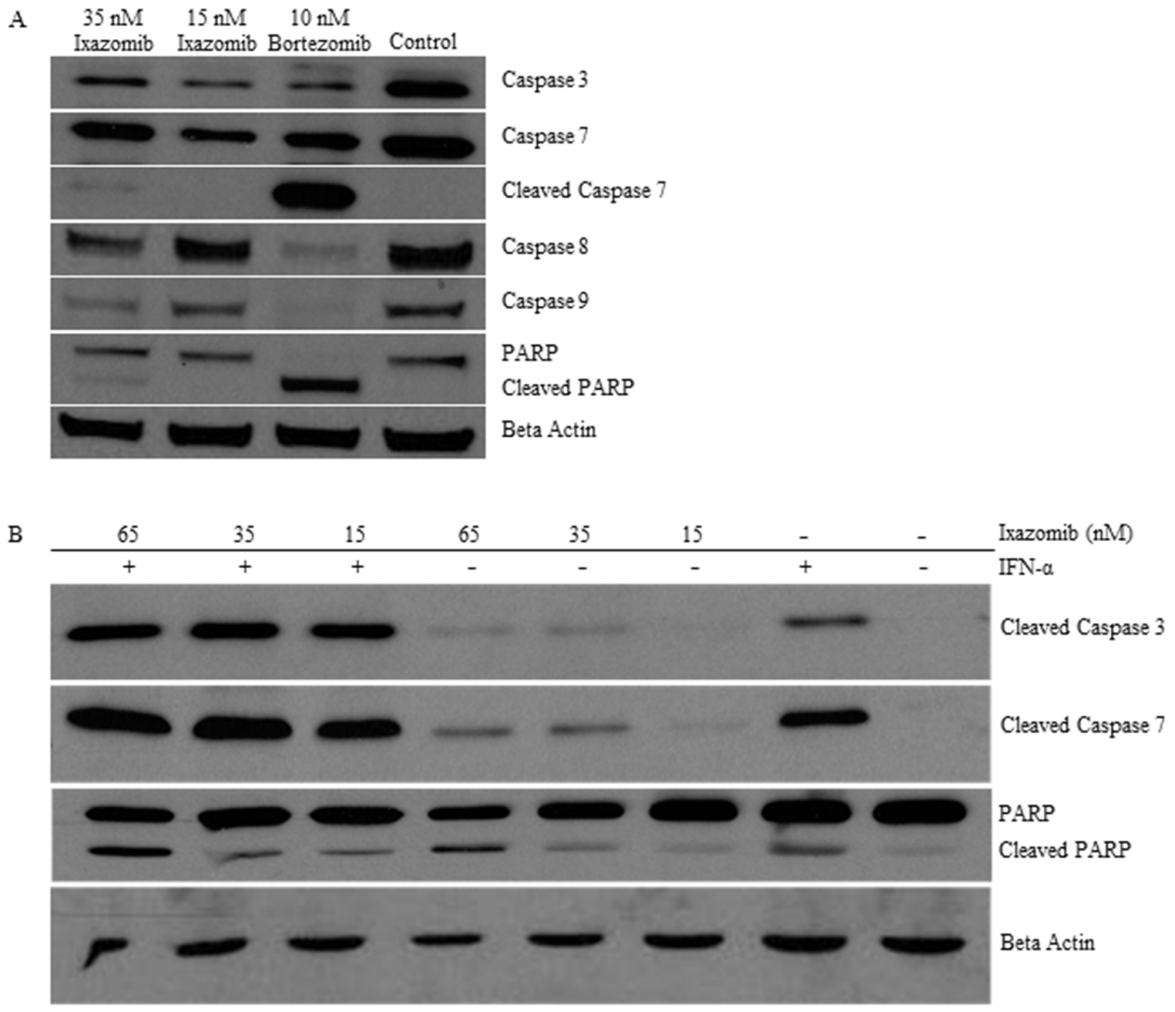
Ixazomib therapy induces processing of effector caspases and poly (ADP-ribose) polymerase (PARP) **A.** BRAF V600E mutant (A375) human melanoma tumor cells plated at a density of 2 × 10^5^ cells/well on a 6 well plate were treated for 48 hours with complete medium supplemented with either 35 nM ixazomib, 15 nM ixazomib, 10 nM bortezomib, or 10% dimethyl sulfoxide (control). Immunoblots were prepared and probed with antibodies specific for caspase-3, caspase-7, cleaved caspase-7, caspase-8, caspase-9, poly(ADP-ribose) polymerase (PARP), and cleaved PARP. **B.** A375 tumor cells were treated for 48 hours with IFN-α (10^4^ units/mL), ixazomib (15-65 nM), or both agents combined and evaluated by immunoblot analysis for cleaved caspase-3, caspase-7, and PARP. Membranes were probed with an anti-β-actin antibody as a loading control.

BRAF V600E mutant (A375) human melanoma tumor cells were plated at a density of 2 × 10^5^ cells/well on a 6 well plate and treated for 48 hours with complete medium supplemented with either 35 nM ixazomib, 15 nM ixazomib, 10 nM bortezomib, or 10% DMSO. Immunoblots were prepared and probed with antibodies specific for caspase-3, caspase-7, cleaved caspase-7, caspase-8, caspase-9, PARP, cleaved PARP, or β-actin as a loading control. Enhanced processing of the major effector caspases (caspase-3, caspase-7, caspase-8 and caspase-9) to their active forms and cleavage of PARP (a target of activated effector caspases) was observed at 48 hours following treatment of the A375 human melanoma cells with ixazomib and bortezomib (Figure 8A). The A375 human melanoma cell line was then treated for 48 hours with IFN-α (10^4^ units/mL), ixazomib (15-65 nM), or both agents combined and evaluated by immunoblot analysis for cleaved PARP, caspase-3 and caspase-7 (Figure 8B). Enhanced processing of caspase-3, caspase-7 and cleavage of PARP was observed following combination treatment of ixazomib and IFN-α.

### Ixazomib also enhances the apoptotic effects of other anti-tumor agents

**Figure 9:**
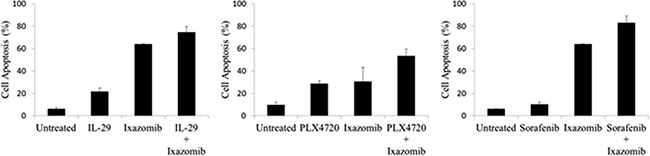
Ixazomib enhances the apoptotic effects of other anti-tumor agents BRAF V600E mutant (A375) human melanoma tumor cells plated at a density of 2 × 10^5^ cells/well on a 6 well plate were treated for 48 hours with 35 nM ixazomib in combination with 100 ng/mL IL-29, 1 μM PLX4720, or 5 μM sorafenib and evaluated for levels of apoptosis via annexin V/propidium iodide staining and flow cytometric analysis. Data represented as mean ± standard error of the mean.

Given the synergistic induction of apoptosis observed with the combination of ixazomib and IFN-α, an effort was made to identify other anti-tumor agents that might demonstrate similar results with ixazomib. Ixazomib was therefore tested in combination with IL-29 (a type III interferon), PLX4720 (a BRAF inhibitor), and sorafenib (a multi-kinase inhibitor). The A375 human melanoma cell line was treated for 48 hours with ixazomib (35 nM) in combination with IL-29 (100 ng/mL), PLX4720 (1 μM), or sorafenib (5 μM) and evaluated for levels of apoptosis as described previously (Figure 9). Each combination treatment resulted in enhanced tumor cell apoptosis as compared to either treatment alone. Combination treatment of IL-29 and IFN-α resulted in an increased level of apoptotic cell death compared to treatment with ixazomib alone (74.4 ± 5.2% vs. 64.1 ± 6.5%, respectively) and IL-29 alone (74.4 ± 5.2% vs. 21.7 ± 3.0%, respectively). The combination treatment of PLX4720 and IFN-α resulted in an increased level of apoptotic cell death compared to treatment with ixazomib alone (53.3 ± 6.2% vs. 30.6 ± 12.5%, respectively) and PLX4720 alone (53.3 ± 6.2% vs. 28.7 ± 2.7%, respectively). Similarly, combination treatment with sorafenib and IFN-α resulted in an increased level of apoptotic cell death compared to treatment with ixazomib alone (82.9 ± 6.0% vs. 64.1 ± 5.5%, respectively) and sorafenib alone (82.9 ± 6.0% vs. 10.3 ± 2.0%, respectively).

### Combination therapy with ixazomib and IFN-α results in enhanced antitumor activity compared with either agent alone in a xenograft model of human melanoma

**Figure 10:**
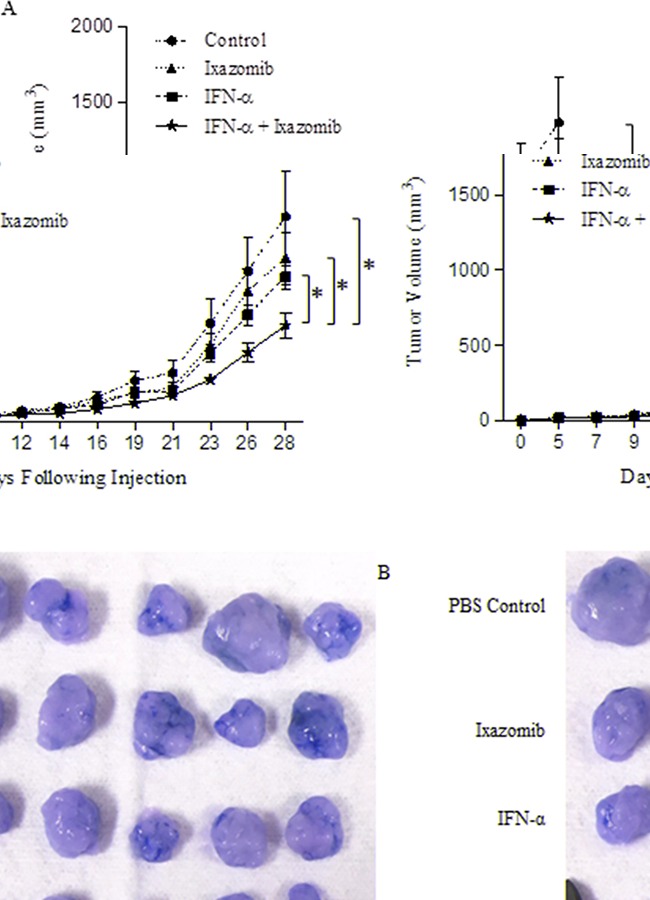
Combination treatment with IFN-α-2b and ixazomib reduces tumor volume *in vivo* Female Balb/c nu/nu (athymic) mice were injected s.c. in the right flank with 2 × 10^6^ human A375 melanoma cells (day 0). Once tumors were palpable, mice were randomized to one of four treatment groups: 1- PBS (n = 11), 2- IFN-α-2b (2 × 10^4^ units/day, intraperitoneal injection; n = 12), 3- ixazomib (7.0 mg/kg twice weekly, oral gavage; n = 12), or 4- IFN-α-2b and ixazomib combined (n = 13). **A.** Bidimensional tumor measurements were obtained three times weekly using microcalipers. Data represented as mean ± standard error of the mean. **B.** Representative image of tumor samples collected at the completion of the study.

The effect of ixazomib in combination with IFN-α was evaluated in an *in vivo* model. Balb/c nu/nu (athymic) mice bearing A375 human melanoma tumors were treated with vehicle, IFN-α-2b (2 × 10^4^ units/day, intraperitoneal injection), ixazomib (7.0 mg/kg twice weekly, oral gavage), or IFN-α-2b and ixazomib combined. Combination treatment with IFN-α-2b and ixazomib demonstrated a significant reduction in tumor volume when compared to vehicle (p = 0.005) and single therapy ixazomib (p = 0.017) and IFN-α-2b (p = 0.036) (Figure 10).

## DISCUSSION

Ixazomib is a novel, orally available selective inhibitor of the 20S proteasome subunit with improved pharmacodynamics and pharmacokinetics compared to bortezomib. Ixazomib has a six-fold faster proteasome disassociation half-life compared to bortezomib, resulting in a faster dissociation from red blood cells and more rapid tumor entry [11, 17, 23]. Ixazomib has also been shown to have stronger antitumor activity and greater proteasome inhibition in tumor tissues compared to bortezomib [16, 17]. Ixazomib is the biologically active moiety of MLN9708 (ixazomib citrate). Ixazomib citrate is available in a capsule format that is rapidly hydrolyzed to ixazomib under physiological conditions [21]. These unique features of ixazomib may help maintain optimal serum concentrations and improve patient convenience and compliance with treatment regimens [23]. The purpose of this study was to examine the antitumor potential of single-agent ixazomib, compare it to bortezomib, and evaluate ixazomib combination treatment with IFN-α in a variety of human melanoma cell lines (A375, WM1366, and MeWo) and in an *in vivo* xenograft model of human melanoma. A secondary aim was to evaluate the usefulness of this combination in BRAF V600E mutant compared to BRAF wild-type melanoma cell lines. We hypothesized that ixazomib would induce apoptosis in human melanoma cells and that combination treatment with IFN-α would enhance its apoptotic activity *in vitro* and reduce tumor volume *in vivo*.

Ixazomib treatment induced apoptotic cell death in both BRAF wild-type and BRAF V600E mutant melanoma cell lines. A slight increase in the efficacy of ixazomib-induced apoptotic cell death was observed in the BRAF V600E mutant (A375) melanoma cell line compared the BRAF wild-type (WM1366) cell line. Combination treatment with ixazomib and IFN-α resulted in a significant, and synergistic, increase in apoptotic cell death in both the BRAF V600E mutant (A375) and the BRAF wild-type (WM1366) melanoma tumor cell lines. Although both cell lines demonstrated a significant increase in apoptotic cell death, the BRAF V600E mutant cell line appeared to have a greater apoptotic response to ixazomib compared to the BRAF wild-type cell line. This may be related to the continuous tumor cell activation of the Raf/MEK/ERK pathway with subsequent sensitization to proteasome inhibition [24, 25].

Combination treatment with ixazomib and IFN-α resulted in a greater decrease in cell proliferation than either agent alone in BRAF V600E mutant (A375) melanoma cell line. However, no synergy was seen between the two treatments with the addition of IFN-α only having minimal effect on cell proliferation. BRAF wild-type (WM1366 and MeWo) human melanoma cell lines were more resistant to ixazomib treatment with no significant decrease in cell proliferation from ixazomib with or without IFN-α treatment. The WM1366 cell line carries an NRAS mutation while the MeWo cell line does not, indicating that the resistance to ixazomib induced inhibition of cell proliferation in BRAF wild-type cell lines might not be mediated by the cell's ability to generate GTP bound active Ras (an upstream activator of the Raf/MEK/ERK kinase cascade), but rather some other aspect of their BRAF wild-type status [25].

Enhanced processing of major effector caspases (caspase-3, caspase-7, caspase-8 and caspase-9) to their active forms and cleavage of PARP (a target of activated effector caspases) was observed at 48 hours following treatment of the BRAF V600E mutant (A375) human melanoma cell line with ixazomib. Additionally, **e**nhanced processing of caspase-3, caspase-7 and cleavage of PARP was observed following treatment of the A375 human melanoma cell line with combination treatment of ixazomib and IFN-α. Our group has previously shown a similar pattern of caspase activation and cleavage of PARP with bortezomib in human melanoma cell lines which demonstrated bortezomib-induced apoptosis as a result of FADD-induced caspase-8 activation [4]. These similar findings suggest that ixazomib and bortezomib may both initiate tumor cell death through activation of the extrinsic pathway of apoptosis via FADD-induced caspase-8 activation.

Similar to the present results, a previous study evaluating the activity of proteasome inhibitors in BRAF V600E mutant colorectal cancer models also demonstrated that BRAF mutant cells were preferentially sensitive to treatment with proteasome inhibitors compared to BRAF wild-type cells [24]. They further evaluated this effect with the addition of BRAF V600E blockade, which reversed the cell's sensitivity to the proteasome inhibitors such that the treatment effects were negated [24]. BRAF V600E mutation results in continuous activation of B-raf and downstream activation of MEK and ERK resulting in the activation of several transcription factors and accumulation of cell cycle proteins. BRAF V600E mutant cells may therefore become dependent on the ubiquitin–proteasome system proteolytic degradation and turnover of these excess proteins. Proteasome inhibition results in the stabilization and continued excess accumulation of cell cycle proteins, which can lead to the activation of anti-proliferative signals, cell cycle disruption, activation of apoptotic pathways, and cell death [3, 4, 24].

These findings suggest that the continuous BRAF V600E gene expression may lead to cell sensitization to treatment with proteasome inhibitors. This issue is likely responsible for the differences in ixazomib sensitivity between the BRAF mutant and BRAF wild-type melanoma cell lines used in this study. This could also be an important factor affecting the results from the phase II clinical trial conducted using bortezomib for the treatment of metastatic melanoma, which demonstrated no clinical anti-tumor activity, since BRAF status was not taken into account [2]. Several other clinical trials demonstrating lack of sufficient clinical activity of bortezomib in patients with metastatic melanoma have also failed to take BRAF mutation status into account (NCT00288041, NCT00580320, NCT01462773, and NCT01078961) [6, 8–10]. Thus, a review of these trial results is in order.

In addition to these clinical trials not taking BRAF mutation status into account, clinical anti-tumor activity may not have been seen as a result of the pharmacokinetic properties of bortezomib. The dosing schedule of bortezomib, for all but one of these clinical trials, was a once weekly with a dosing interval of 168 hours [6, 8–10]. The mean elimination half-life of bortezomib upon multiple dosing ranges from 40 to 193 hours after the 1 mg/m^2^ dose and 76 to 108 hours after the 1.3 mg/m^2^ dose. With this long dosing interval and broad elimination half-life it is possible that these patients were unable to truly obtain optimal drug treatment levels and a steady state. On the other hand, the mean elimination half-life of ixazomib is much longer with a half-life of 228 days. Therefore, the longer elimination half-life of oral ixazomib makes it a promising agent for a combined treatment of BRAF V600E mutant melanoma with ixazomib and IFN-α.

To our knowledge, this is the first study evaluating the use of an orally available proteasome inhibitor (ixazomib) in the treatment of melanoma. In this study, the apoptotic effects of ixazomib with or without IFN-α were evaluated in melanoma. A similar study performed by our group had previously evaluated the use of bortezomib (intravenously available proteasome inhibitor) with or without IFN-α in melanoma with similar findings [4]. Additionally, this study has demonstrated a significant reduction in tumor volume in an *in vivo* xenograft model of human melanoma with combination treatment of IFN-α-2b and ixazomib when compared to vehicle and single therapy ixazomib or IFN-α-2b. The results from this study, in addition to previous supporting studies, demonstrate the potential for further studies of a melanoma treatment regimen using ixazomib in combination with IFN-α. It is possible that the improved pharmacodynamics and pharmacokinetics of ixazomib, compared to bortezomib, will result in improved anti-tumor activity in melanoma. Previous studies have demonstrated that ixazomib has a shorter proteasome dissociation half-life, a larger blood volume distribution at a steady state, and a greater and more constant biodistribution than bortezomib [4, 14, 17]. In addition, previous clinical trials of orally administered ixazomib citrate for the treatment of multiple myeloma have demonstrated improved patient tolerability and a safer toxicity profile compared to bortezomib (NCT00963820 and NCT00932698) [18, 19]. Ixazomib citrate is currently being tested in multiple phase III clinical trials for the use in hematologic malignancies [11, 15].

Together these pre-clinical and clinical data suggest that combined treatment with ixazomib and IFN-α represents a novel treatment strategy for inducing synergistic apoptotic tumor cell death in BRAF V600E mutant melanoma. Further delineation of the exact mechanism of cell death activating pathways induced by proteasome inhibitors and the mechanisms of proteasome inhibitor resistance by BRAF wild-type melanoma may help identify future therapeutic anti-tumor molecular targets.

## MATERIALS AND METHODS

### Materials

The A375 human melanoma cell line was purchased from the American Type Culture Collection (ATCC Manassas, Virginia). The WM1366 and MeWo cell lines were obtained from Dr. Saldano Ferrone (Massachusetts General Hospital, Boston, MA). Ixazomib (MLN2238) and bortezomib (Velcade, PS-341) were obtained from Millennium Pharmaceuticals, Inc. (Cambridge, MA). Recombinant human IFN-α was obtained from Schering-Plough, Inc. (Kenilworth, NJ).

### Analysis of apoptosis via annexin V/Propidium Iodine (PI) staining

Apoptosis-induced phosphatidyl serine exposure was measured in tumor cells by flow cytometric analysis on an LSR II flow cytometer (BD Pharmingen, San Jose, CA) using APC-conjugated anti-annexin V and PE-conjugated anti-propidium iodide (BD Pharmingen, San Jose, CA) as previously described [26]. Each analysis was performed utilizing at least 10,000 cellular events. The percentages of positively staining cells were calculated within each treatment group through flow cytometric analysis (FlowJo, Ashland, OR).

### Confocal microscopy

Differential interference contrast (DIC) images were obtained on an Olympus Fluoview 1000MPE confocal microscope using LUMPLFL 10XW (N.A. 0.3) and 40XW (N.A. 0.8) objectives. All images were processed using Olympus Fluoview (v.2.1b) software.

### Proliferation assays

The proliferation of melanoma cells treated with ixazomib with or without IFN-α was evaluated using the methylthiazolyldiphenyl-tetrazolium bromide (MTT) Cell Proliferation Assay (ATCC ® 30-1010K, Manassas, VA) and optical density (O.D.) recorded at a 570 nm wavelength using a microtiter plate reader as previously described with modification [27].

### Trypan blue staining

Cell viability was evaluated by trypan blue staining. An aliquot of 1 × 10^5^ cells was used for 1:1 staining with 0.4% trypan blue. Cells were incubated in the trypan blue stain for 3 minutes and were then immediately analyzed. The number of viable and dead cells was determined using a hemocytometer.

### Immunoblot analysis

Immunoblots were prepared and probed with antibodies specific for caspase-3, caspase-7, caspase-8, caspase-9, cleaved caspase-3, cleaved caspase-7, poly(ADP-ribose) polymerase (PARP) (Cell Signaling Technology, Danvers, MA), or β-actin (Sigma, St. Louis, MO). Following incubation with the appropriate horseradish peroxidase-conjugated secondary antibody, immune complexes were detected using an enhanced chemiluminescence detection kit (Thermo Scientific, Waltham, MA) and analyzed by quantitative densitometry using Optimas 6.51 image analysis software (Media Cybernetics, Carlsbad, CA).

### Murine tumor model and treatments

The effect of ixazomib in combination with IFN-α was evaluated using a xenograft model of human melanoma. In brief, female Balb/c nu/nu (athymic) mice (Taconic Farms, Inc.) were injected s.c. in the right flank with 2 x 10^6^ human A375 melanoma cells (day 0). Once tumors were palpable, mice were randomized to one of four treatment groups: (a) PBS, (b) IFN-α-2b (2 x 10^4^ units/day, intraperitoneal injection), (c) ixazomib (7.0 mg/kg twice weekly, oral gavage), or (d) IFN-α-2b and ixazomib combined. Treatment groups consisted of 11-13 mice/group. Bidimensional tumor measurements were obtained three times weekly using microcalipers. At the conclusion of the study, the mice were sacrificed and the tumor samples from each mouse were resected.

### Statistical analysis

The linear mixed effects model was employed to model assay data with experiments as random effects. Comparisons were performed across treatment groups using t-statistics. Multiplicity was adjusted across cell lines. Unadjusted p-values were provided for each treatment pair comparison and also for the interaction between IFN-α and ixazomib. Holm-Bonferroni adjusted p-values for synergy effects were provided. Significance was set at the 0.05 level. For the *in vivo* animal experiment to investigate the drug effects on tumor growth inhibition, the tumor volume was measured over time for each mouse. As the observations from the same animal within the same cage are correlated, a linear mixed effects model was used to take account of the correlation among those observations. The tumor growth rate as well as the mean tumor volume averaged across time was compared between the ixazomib plus IFN-α combination treatment group and the single agent treatment regimens. Holm's procedure was used to control for multiple comparisons. Adjusted p-value < 0.05 was considered as significant.
